# Can lung ultrasound replace chest computed tomography in pediatric patients with complicated community-acquired pneumonia?

**DOI:** 10.1186/s13052-025-02168-4

**Published:** 2025-12-23

**Authors:** Erini Farid Fawzy, Asmaa Mahmoud Hamed, Sara Mahmoud Kamel, Noussa Ragab Mohamed, Iman Hassan Deraz, Aya Samir Mohamed

**Affiliations:** 1 Department of Pediatrics, Faculty of Medicine, Cairo University, Cairo, Egypt; 2 Department of Pediatric Radiology, Faculty of Medicine, Cairo University, Cairo, Egypt

**Keywords:** Complicated community-acquired pneumonia, Lung ultrasound, Pediatric, Computed tomography, Pleural effusion, Lung consolidation, Diagnostic imaging

## Abstract

**Background:**

Complicated community-acquired pneumonia (CCAP) in children can result in severe morbidities. While computed tomography (CT) is the gold standard for diagnosis, its radiation exposure has led to the increased use of lung ultrasound (LUS) as a safer radiation-free alternative. This study aimed to evaluate the diagnostic accuracy of LUS in detecting complications in pediatric patients with CCAP and determine its value in their follow up as well as its value in determining patients liable for surgical intervention as pleural decortication comparing its efficacy with chest CT.

**Methods:**

This is a prospective observational cohort study that was conducted on 56 pediatric patients with CCAP at our tertiary-level referral pediatric hospital. Patients underwent clinical evaluation, laboratory investigations, chest X-ray, CT, and LUS. The sensitivity, specificity, and accuracy of LUS were compared with CT for detecting consolidations, pleural thickening, effusions, lung abscesses, and hydropneumothorax.

**Results:**

Compared to CT chest, LUS showed high sensitivity (92.6% for the right lung and 94.1% for the left lung) and specificity (88% and 100%, respectively) in detecting pleural effusions and consolidations, with substantial agreement with CT (*p* < 0.001). However, it had lower sensitivity in detecting lung abscesses (33.3% for the right lung, 0% for the left lung) and hydropneumothorax. Pleural thickness measured by LUS was a predictor for surgical intervention (cut-off > 2.2 mm). Follow-up LUS indicated significant improvement in lung lesions after one month.

**Conclusion:**

LUS is a reliable tool for detecting pleural effusions and consolidations in pediatric CCAP, reducing the need for CT in many cases. However, its limitations in identifying abscesses and hydropneumothorax highlight the need for combined diagnostic approaches.

## Background

Community-acquired pneumonia (CAP) remains a leading cause of morbidity and mortality in children worldwide, especially in developing countries where access to advanced medical diagnostics can be limited [1]. Traditionally, the diagnosis and management of complicated CAP have relied heavily on clinical assessment and chest radiography. However, these methods have limitations, such as low sensitivity in detecting certain complications like pleural effusion or abscess formation [2]. Recently, chest sonography, or lung ultrasound (LUS), has emerged as a promising alternative diagnostic tool due to its non-invasiveness, lack of ionizing radiation, portability, and high diagnostic accuracy for pediatric lung pathologies [3].

The utility of chest sonography in diagnosing and managing complicated CAP in children has gained considerable attention in recent years. Studies have demonstrated its efficacy in identifying pneumonia-related complications, such as parapneumonic effusions, necrotizing pneumonia, and pneumothorax, which are often missed or underestimated on standard chest radiographs [4, 5]. Moreover, LUS has proven to be highly beneficial for follow-up assessments, enabling clinicians to monitor disease progression and therapeutic responses more dynamically than with traditional radiography [6]. Despite its increasing adoption, questions remain regarding its standardization, training requirements, and integration into clinical pathways for pediatric CAP management.

Furthermore, emerging evidence suggests that chest sonography can significantly reduce the time to diagnosis and the length of hospital stay for pediatric patients with complicated CAP, which is crucial in resource-limited settings where prolonged hospitalization can strain healthcare resources [7]. The ability of LUS to provide real-time visualization of lung pathology allows for quicker decision-making regarding the need for advanced interventions, such as thoracentesis or chest tube placement, potentially leading to better outcomes and reduced healthcare costs [8].

This cohort aims to explore the role of chest sonography in the diagnosis and follow-up of complicated CAP in children, examining recent advancements, current practices, and the challenges associated with its implementation.

## Methods

### Study design

This prospective observational cohort study included 56 pediatric patients diagnosed with complicated CAP, admitted to inpatient wards of the Children’s Hospital of Cairo University, over a period of one year from May 2023 to June 2024. Ethical considerations were paramount with the research undergoing review by the Scientific Ethical committee of Cairo University Hospital and informed written consent was collected from all participants (Code: MS-132-2023).

### Subjects

A total of 56 pediatric patients referred to our hospital with complicated community acquired pneumonia (CAP). Complicated CAP in this context is defined by both local complications (including: para pneumonic effusion, empyema, hydro pneumothorax, lung abscess and necrotizing pneumonia) and systemic complications (including bacteremia, multiorgan failure, and acute respiratory distress syndrome) in previously healthy children [9].

Inclusion criteria included children aged 2 months to 13 years of both genders diagnosed with complicated CAP, based on physical and radiographic findings.

Exclusion criteria included patients with underlying chronic or congenital lung diseases (e.g. patients with cystic fibrosis, congenital cystic adenomatoid malformation), those who had pleural effusion due to autoimmune diseases, malignancies, or other systemic conditions and cases of hospital-acquired pneumonia.

### Sample size

The sample size was calculated using the epi-info software program, with confidence level of 95% and a margin of error 5% based on the estimated prevalence of community-acquired pneumonia to be 40/1000 (Bloise et al., 2021) based on the following equation:

- Sample size (n) = [DEFF*Np(1-p)]/ [(d2/Z21-α/2*(N-1) + p*(1-p).
- The sample size was calculated to be at least 56 patients.

### Methodology

All participants underwent comprehensive clinical assessments, including a detailed medical history and physical examination. Particular attention was given to demographic details, disease onset, symptomatology (such as dyspnea, fever, cough, and chest pain), and prior antibiotic use. Management strategies, including conservative treatment, chest tube insertion, and any surgical interventions, were documented.

Clinical examinations focused on vital signs, respiratory distress, oxygen saturation, and a thorough chest evaluation for signs such as wheezes or crepitations. Laboratory investigations included a complete blood count, C-reactive protein, ESR, and blood cultures. For those with pleural involvement, thoracentesis was performed to analyze and culture pleural fluid.

Chest X-ray was done to assess lung volume, opacity, and any presence of effusions or air-fluid levels. For a more detailed evaluation, a non-contrast chest CT was conducted to identify areas of consolidation, collapse, effusion, or abscess formation. The CT-chest results were interpreted by independent, experienced consultants of radiology at the department of pediatric radiology Cairo University.

We performed LUS for each case twice; within five days of admission (with total number of 56 cases) and follow up after 1 month (with total number of 55 cases; one patient was missed in the follow up LUS). at the radiology department by a single experienced pediatric radiologist with 4-year experience in lung ultrasound, blinded by the findings in the CT done, using a standardized four-view protocol to assess pleural thickening, effusion, consolidation, and cavities. The patient was asked to lie in the supine position to assess the apical and anterior segments of the upper and lower lobes, then he/she was turned on both lateral decubitus positions to assess the posterior segments. LUS scoring was based on aeration patterns to gauge the severity of lung involvement, following the method outlined by Oulego et al. [10].

The agreement between the findings in the CT-chest and the initial (first) lung ultrasound was interpreted and analyzed.

**Fig. 1:**
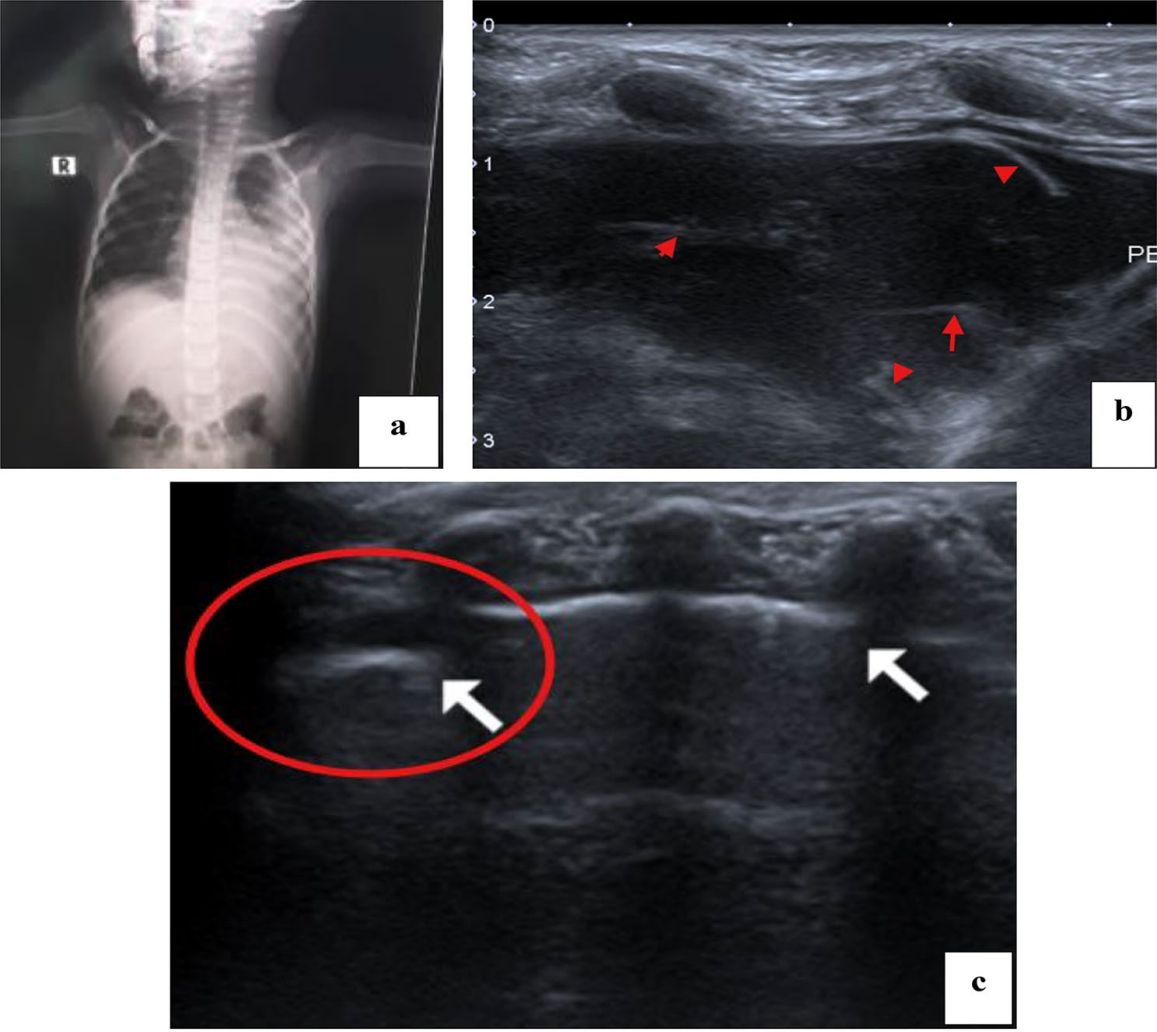
A 5-year-old female child with complicated community-acquired pneumonia. (**a**) The initial plain x-ray done on admission showed an obliterated costophrenic angle with the opacification of the lower part of the left hemithorax. (**b**) Lung ultrasound examination done with the probe placed at the left costophrenic angle showing moderate pleural collection/effusion with internal echogenic bands (the arrows represents the bands). (**c**) Lung ultrasound examination with the probe placed at zone 3 of the left lung, showing transition point on the lung surface where the lower arrow represents normal lung sliding, compared with absent lung sliding, the arrow within the oblong, lung point sign, denoting presence of pneumothorax

**Fig. 2:**
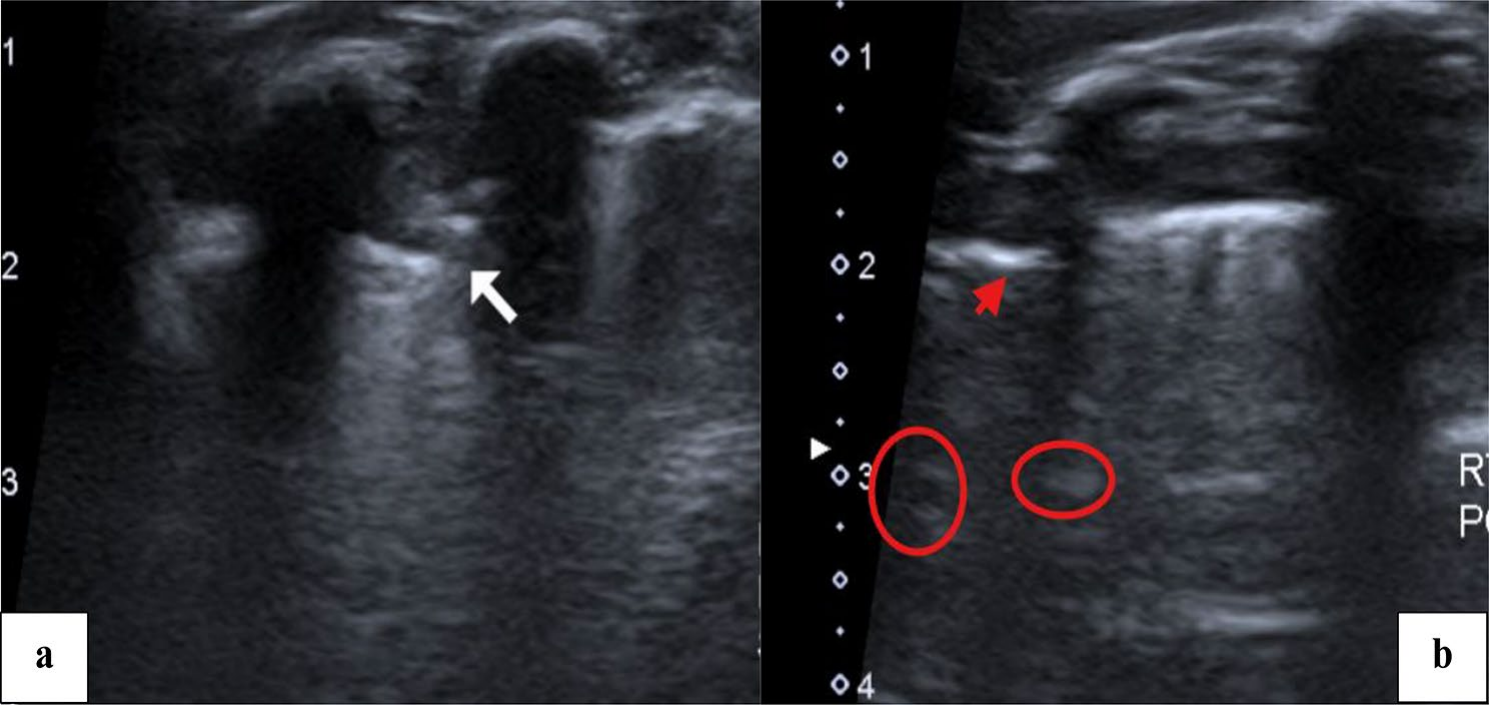
A 3-month-old female child with complicated community-acquired pneumonia. (**a**) Lung ultrasound examination done with the probe placed at the right lung, zone 1 showing loss of A-lines with consolidation of the lung, pleural thickening, and irregularity, arrow. (**b**) Lung ultrasound examination with the probe placed at zone 3 of the right lung showing transition point on the lung surface where the arrow represents absent lung sliding, denoting presence of pneumothorax. Fluid bronchogram is also noted (circles)

**Fig. 3:**
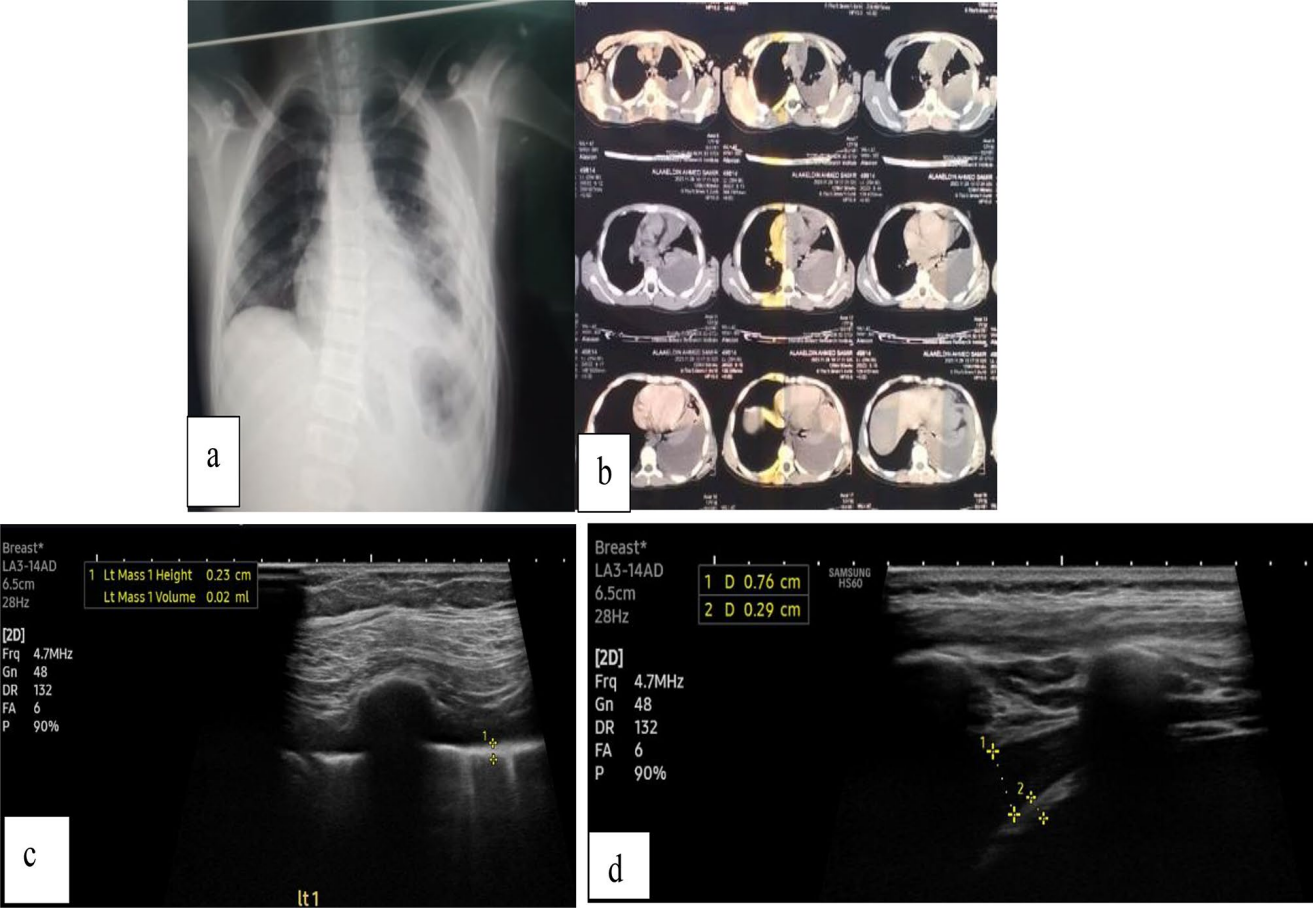
9 years old male boy presented by shortness of breath and diagnosed as CAP .(**a**) Plain chest x-ray PA view showing obliterated left costophrenic angle by density rising towards the axilla and left lateral density.(**b**) Non contrast CT chest done on admission showing moderate left sided pleural effusion with left lower lobar lung collapse.(**c**,** d**) Follow up lung U/S showing thickened pleura of the left lung, measuring 2.3–2.9 mm in thickness with still noted mild left sided pleural effusion

**Fig. 4:**
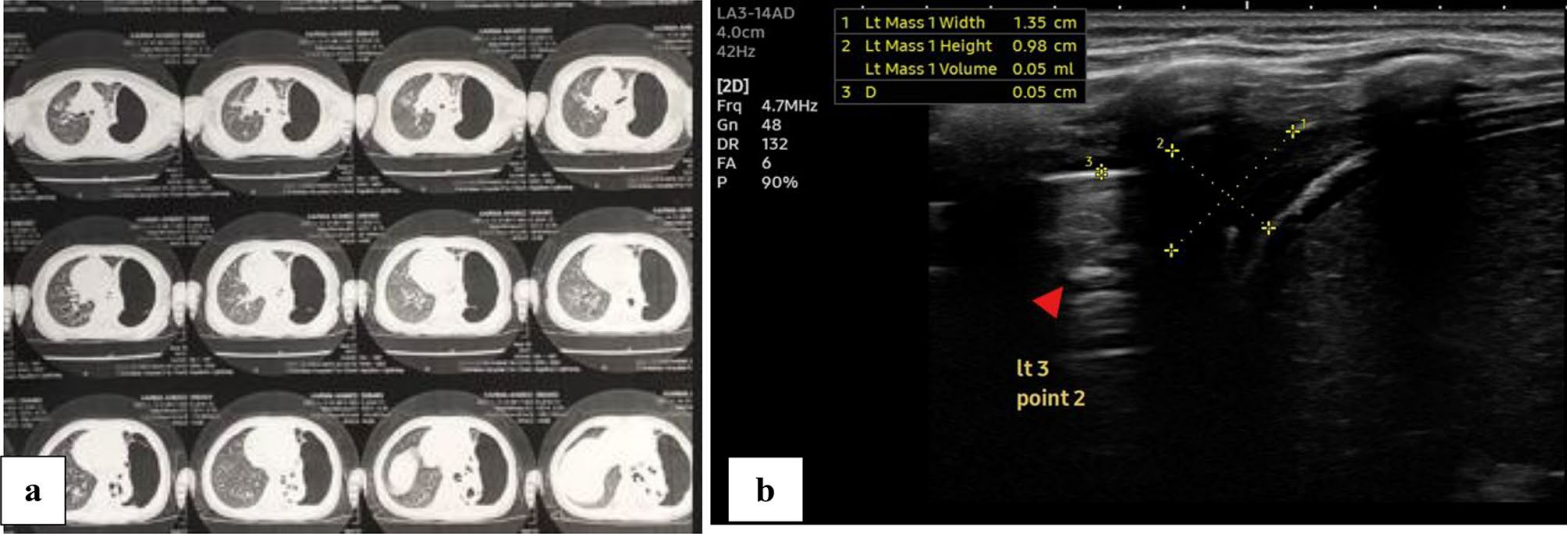
Follow up lung U/S for female patient with complicated community acquired pneumonia. (**a**) The initial CT done on admission of the patient showing moderate encysted left sided pneumothorax with left lower lobar collapse. (**b**) The follow up lung U/S done after chest tube insertion showing residual mild right sided pleural effusion with left lower lobar increased echogenicity, loss of the A lines with fluid bronchogram (arrow head)

**Fig. 5:**
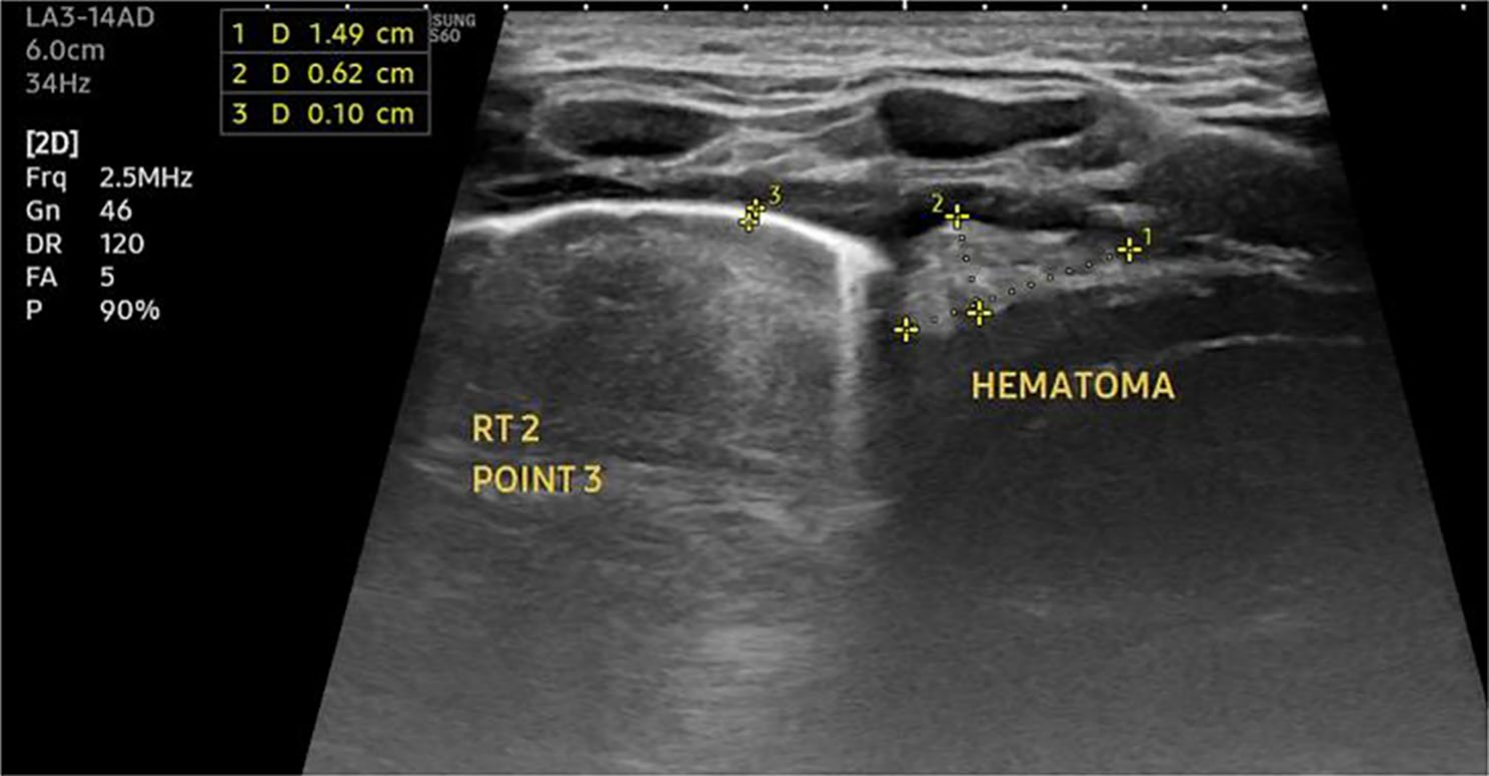
Lung U/S for female patient with complicated community acquired pneumonia showing echogenic hematoma at the right costophrenic recess, pleural thickening, loss of the A lines within the lung with hepatization pattern of pneumonia

Radiological findings in the follow up LUS was compared to findings in the initial LUS. Examples of CT chest, initial and follow up LUS findings are shown in Figs. 1, 2, 3, 4 and 5.

### Statistical analysis

Data were analyzed using IBM SPSS software package version 20.0. (Armonk, NY: IBM Corp) Qualitative data were described using number and percent. The Shapiro-Wilk test was used to verify the normality of distribution Quantitative data were described using range (minimum and maximum), mean, standard deviation, median and interquartile range (IQR). Significance of the obtained results was judged at the 5% level.

## Results

This prospective observational cohort study included 56 pediatric patients diagnosed with complicated CAP. The study cohort consisted of 29 males (51.8%) and 27 females (48.2%), with a median age of 49 months (IQR 36–73 months). The median weight was 17 kg (IQR 11–21 kg), and the median height was 102 cm (IQR 91–115 cm) (Table 1).

**Table 1:** Demographic characteristics of the study population

Demographic data	No.	%
Gender		
Male	29	51.8
Female	27	48.2
**Age (months)**		
Min. – Max.	2.50–144.0	
Mean ± SD.	59.92 ± 36.30	
Median (IQR)	49.0 (36.0–73.0)	
**Weight (kg)**		
Min. – Max.	4.0–48.0	
Mean ± SD.	18.12 ± 8.87	
**Height (cm)**		
Min. – Max.	57.0–157.0	
Mean ± SD.	104.25 ± 21.63	

Demographic data were presented in number (n), percent (%), mean, median and standard deviation. IQR: Inter quartile range SD: Standard deviation

Fever was the most common presenting symptom, occurring in all patients (100%), followed by cough (73.2%), dyspnea (51.8%), abdominal pain (35.7%) and chest pain (12.5%). Clinical examination on presentation showed that all patients exhibited diminished air entry and 71.4% of patients exhibited respiratory distress (Table 2).

**Table 2:** Clinical presentation of the study population

Symptoms	No.	%
Fever	56	100.0
Cough	41	73.2
Chest pain	7	12.5
Dyspnea	29	51.8
Abdominal pain	20	35.7
**Signs**	**No.**	**%**
Respiratory distress	40	71.4
Diminished air entry	56	100
Crepitation	9	16.1
Wheezes	4	7.1

Most patients (53.6%) required chest tube insertion for continuous thoracic drainage, with a median duration of 17 days. Four patients (7.1%) needed decortication following unsuccessful chest tube management, and four required streptokinase injection. The median duration of hospital stay was 21 days (IQR 14-29.5 days) (Table 3).

**Table 3:** Management plan of the study population

Management	No.	%
Conservative medical treatment	22	39.3
Chest tube	30	53.6
Chest tube + decortication	4	7.1
**Need for streptokinase**	4	7.1
**Duration of chest tube (days) (n = 34)**		
Min. – Max.	4.0–40.0	
Mean ± SD.	18.15 ± 9.18	
**Duration of hospital stay (days)**		
Min. – Max.	10.0–42.0	
Mean ± SD.	22.09 ± 8.41	

Agreement between chest CT findings and initial (first) LUS done for each patient was assessed. LUS showed moderate agreement with CT for detecting consolidation (*p* < 0.001) and pleural effusion (*p* < 0.001). LUS demonstrated good specificity for detecting lung collapse (96.3% for the right lung and 100% for the left lung) but lower sensitivity (31.0% for the right lung and 42.1% for the left lung) (Tables 4, 5, 6 and 7).

**Table 4:** Agreement between chest CT and first LUS regarding lung consolidation

The affected lung		CT finding (Consolidation)				Sensitivity	Specificity	PPV	NPV	Accuracy
		Negative		Positive						
		No.	%	No.	%					
**LUS finding (Consolidation)**	**Right**	**(n = 29)**		**(n = 27)**						
	Negative	18	62.1	2	7.4	92.59	62.07	69.44	90.0	76.79
	Positive	11	37.9	25	92.6					
	**κ (Level of agreement)**	0.540(Moderate)								
	**p**	< 0.001^*^								
	**Left**	**(n = 39)**		**(n = 17)**						
	Negative	28	71.8	1	5.9	94.12	71.79	59.26	96.55	78.57
	Positive	11	28.2	16	94.1					
	**κ (Level of agreement)**	0.565(Moderate)								
	**p**	< 0.001^*^								

κ: kappa test p: p value for association between different categories *: Statistically significant at *p* ≤ 0.05 PPV: Positive predictive value NPV: Negative predictive value

**Table 5:** Agreement between chest CT and first LUS regarding collapse

The affected lung		CT finding (Collapse)				Sensitivity	Specificity	PPV	NPV	Accuracy
		Negative		Positive						
		No.	%	No.	%					
**LUS finding (Collapse)**	**Right**	**(n = 27)**		**(n = 29)**						
	Negative	26	96.3	20	69.0	31.03	96.30	90.0	56.52	62.50
	Positive	1	3.7	9	31.0					
	**κ (Level of agreement)**	0.267(Fair)								
	**P**	0.014^*^								
	**Left**	**(n = 37)**		**(n = 19)**						
	Negative	37	100.0	11	57.9	42.11	100.0	100.0	77.08	80.36
	Positive	0	0.0	8	42.1					
	**κ (Level of agreement)**	0.490 (moderate)								
	**P**	< 0.001^*^								

κ: kappa test p: p value for association between different categories *: Statistically significant at *p* ≤ 0.05 PPV: Positive predictive value NPV: Negative predictive value

**Table 6:** Agreement between chest CT and first LUS regarding pleural effusion

The affected lung		CT finding (Effusion)				Sensitivity	Specificity	PPV	NPV	Accuracy
		Negative		Positive						
		No.	%	No.	%					
**LUS finding (Effusion)**	**Right**	**(n = 25)**		**(n = 31)**						
	Negative	22	88.0	1	3.2	96.77	88.0	90.91	95.65	92.86
	Positive	3	12.0	30	96.8					
	**κ (Level of agreement)**	0.854 (Very good)								
	**p**	< 0.001^*^								
	**Left**	**(n = 36)**		**(n = 20)**						
	Negative	36	100.0	0	0.0	100.0	100.0	100.0	100.0	100.0
	Positive	0	0.0	20	100.0					
	**κ (Level of agreement)**	1.000(Very good)								
	**p**	< 0.001^*^								

κ: kappa test p: p value for association between different categories *: Statistically significant at *p* ≤ 0.05 PPV: Positive predictive value NPV: Negative predictive value

**Table 7:** Agreement between chest CT and first LUS regarding lung abscess

The affected lung		CT finding (Abscess)				Sensitivity	Specificity	PPV	NPV	Accuracy
		Negative		Positive						
		No.	%	No.	%					
**LUS finding (Abscess)**	**Right**	**(n = 53)**		**(n = 3)**						
	Negative	53	100.0	2	66.7	33.33	100.0	100.0	96.36	96.43
	Positive	0	0.0	1	33.3					
	**κ (Level of agreement)**	0.486 (Moderate)								
	**p**	<0.001^*^								
	**Left**	**(n = 55)**		**(n = 1)**						
	Negative	54	98.2	1	100.0	0.0	98.18	0.0	98.18	96.43
	Positive	1	1.8	0	0.0					
	**κ (Level of agreement)**	-0.018 (Poor)								
	**p**	0.892								
	**Left**	**(n = 48)**		**(n = 8)**						
	Negative	48	100.0	8	100.0	0.0	100.0	–	85.71	85.71
	Positive	0	0.0	0	0.0					
	**κ (Level of agreement)**	–								
	**p**	–								

κ: kappa test p: p value for association between different categories *: Statistically significant at *p* ≤ 0.05 PPV: Positive predictive value NPV: Negative predictive value

Sensitivity, specificity, PPV, NPV and accuracy of LUS in detection of hydropneumothorax compared to the CT chest were presented in percent

κ: kappa test p: p value for association between different categories Statistically significant at *p* ≤ 0.05 PPV: Positive predictive value NPV: Negative predictive value

In the current study, the first LUS was performed within 5 days of admission, and the follow-up was done after 1 month (Table 8).One patient was missed in the follow up LUS (no.55). There is a statistically significant P value between the first and follow-up LUS (p value ≤ 0.05) regarding consolidation, collapse, presence of effusion, size of effusion if still present, presence of septations, and pleural thickness.

**Table 8:** Comparison between first (*n* = 56) and follow-up (*n* = 55) LUS of the included patients

LUS findings	First LUS (*n* = 56)		Follow up LUS (*n* = 55)		*p*
	No.	%	No.	%	
Consolidation	55	98.2	41	74.5	*p* < 0.001
Collapse	18	32.1	4	7.3	*p* < 0.001
Effusion					
Free effusion	30	53.6	23	41.8	*p* < 0.001
Encysted	11	19.6	3	5.5	
Free + encysted	12	21.4	6	10.9	
Presence of septations	20	35.7	5	9.1	*p* < 0.001
Abscess	2	3.6	0	0.0	*p* = 1.000
Hydropneumothorax	2	3.6	2	3.6	*p* = 1.000
Pleural thickness (mm)					
Min. – Max.	0.50–18.0		0.50–15.0		*p* < 0.001
Mean ± SD.	4.14 ± 3.57		2.55 ± 2.87		
Median (IQR)	3.15(1.95–4.65)		1.40(0.85–2.55)		
Lung sliding					
Normal	53	94.6	51	92.7	*p* = 1.000
Decreased	3	5.4	4	7.3	

Data of first and follow up LUS were presented in number (No) and percent (%)

IQR: Inter quartile range SD: Standard deviation *: Statistically significant at *p* ≤ 0.05

**Fig. 6:**
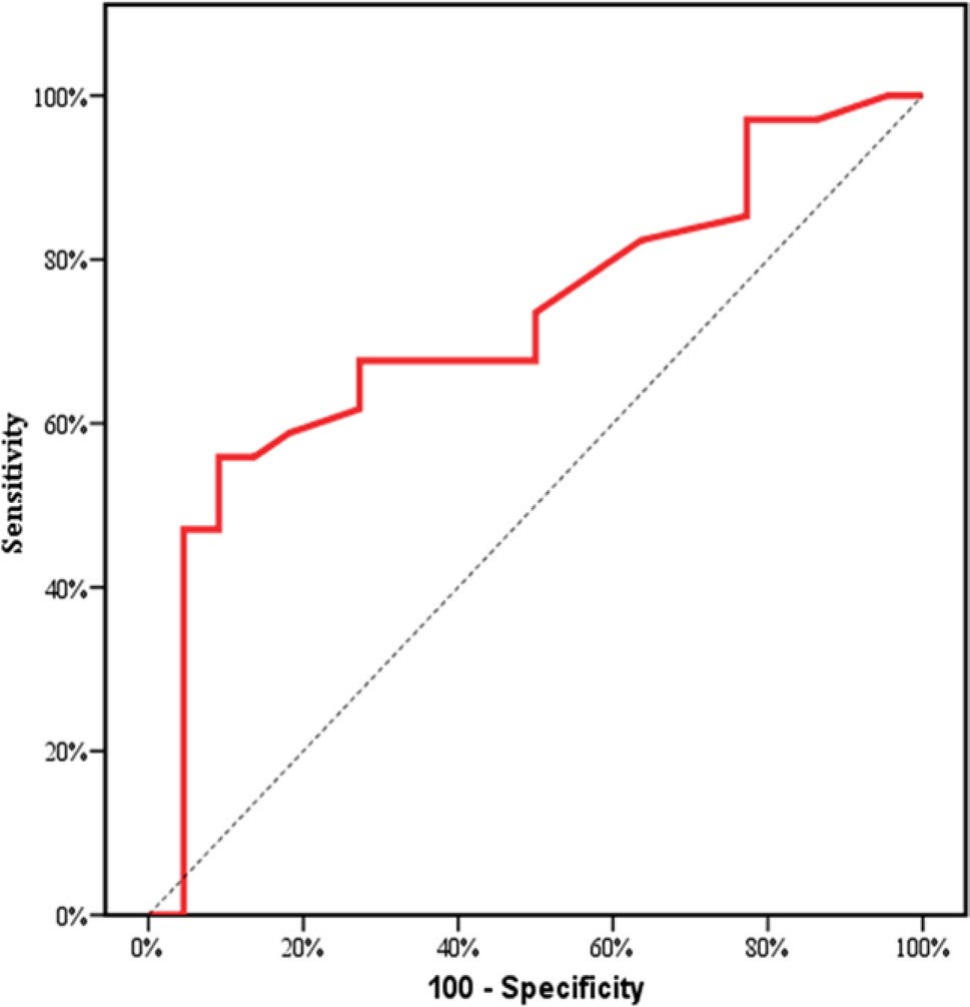
ROC curve for Pleural thickness (mm) to predict the need for surgical intervention

Analysis showed that pleural thickness measured by LUS could predict the need for surgical intervention (cut-off > 2.2 mm; sensitivity 73.5%, specificity 50%) (Fig. 6) .

## Discussion

CAP is defined as the presence of clinical signs and symptoms of pneumonia in a previously healthy child who acquired the infection outside a healthcare setting [11]. While most cases of pediatric CAP resolve with appropriate treatment, some children develop severe local or systemic complications, termed complicated community-acquired pneumonia (CCAP). CCAP is associated with various local complications, including parapneumonic effusion, empyema, necrotizing pneumonia, and lung abscess [12].

This study aimed to investigate the role of LUS compared to CT chest in guiding the diagnosis and management as well as assessing the clinical and imaging characteristics of pediatric patients with CCAP. Our cohort consisted of 56 patients with a near-equal gender distribution and a median age of 49 months, aligning with the demographic characteristics reported by Ionescu et al. [11]. Additionally, 30.4% of our patients had a body weight below the 10th percentile, similar to the proportion identified in the study of Tuğcu et al. [13] demonstrating that host characteristics including nutritional status may not significantly predispose children to CCAP, unlike the virulence of the causative organisms.

The clinical presentation of our cohort highlighted fever as the most common symptom, followed by cough and dyspnea. This finding is consistent with the literature, where fever is reported in nearly all cases of pediatric CAP [14, 15]. Notably, abdominal pain was observed in 35.7% of our patients, and in three cases, it was the sole presenting symptom alongside fever. These patients were initially misdiagnosed with surgical conditions, such as appendicitis or renal stones. This underscores the diagnostic challenge posed by referred pain due to diaphragmatic irritation or mechanisms similar to mesenteric adenitis [16, 17]. Thus, clinicians should maintain a high index of suspicion for CCAP in children presenting with atypical symptoms like abdominal pain to avoid misdiagnosis. According to Krenke et al., abdominal pain was also a common presentation (30.3%) in children with pleural empyema and para pneumonic pleural effusion [14].

The majority of patients (71.4%) presented with respiratory distress without desaturation. This is consistent with Lahti et al. [15] and Sakran et al. [18] where respiratory distress was present in 78% and 83% of cases respectively. The presence of diminished air entry on auscultation in all our patients reinforces its importance as a clinical sign of CCAP, consistent with the study of Yavuz et al. [19]. However, crepitations were detected in only 16.1% of our patients, and this is consistent with the findings of Lahti et al. [15]. In our opinion, the low percentage of crepitations in CCAP may be explained with the presence of effusion causing lung collapse, thereby masking the typical sounds associated with pneumonia.

In many developing countries, CT chest remains a routine imaging modality for characterizing pleural effusions and underlying parenchymal disease before chest tube placement or surgery. However, the incremental use of CT in pediatric age raises concerns about radiation exposure, with risks as high as one in 500 for radiation-induced malignancy [20]. Our study aimed to explore the role of LUS as an alternative imaging strategy.

Our findings indicate that LUS is a highly sensitive tool for detecting right and left lung consolidations, with sensitivities of 92.6% and 94.1%, respectively. However, its specificity was lower (62.1% for the right lung and 71.3% for the left lung), as LUS cannot easily differentiate between consolidation and other pathologies, such as lung collapse [21]. These results are consistent with the study of Yan et al. [22] in which lung ultrasound displayed 90.6% sensitivity and 66.1% specificity compared with chest CT in the detection of consolidation, as well as the prospective clinical study performed by Cortellaro et al. [23] who found that the sensitivity of chest ultrasound in the detection of pneumonia compared to CT chest is 96%. While LUS offers a non-invasive and radiation-free alternative, its limitations, such as operator dependency and reduced specificity, should be considered when interpreting findings.

For detecting pleural effusion, LUS showed excellent sensitivity (96.8% for the right lung and 100% for the left lung) and specificity (88% and 100%, respectively), with high overall accuracy. These results align with meta-analyses conducted by Hansell et al. [24] who compared the accuracy of lung ultrasound (LUS) to the CXR and CT for the diagnosis of pleural effusion and found a similar result to our study where LUS had a pooled sensitivity of 91% and a pooled specificity of 92%. Thus, LUS could serve as an effective primary imaging tool in diagnosing pleural effusions in pediatric CCAP, minimizing the need for CT and reducing radiation exposure.

Regarding air containing lesions; lung abscesses and hydropneumothorax, LUS showed lower sensitivity. The specificity of LUS for detecting lung abscesses was high (100% for the right lung and 98.2% for the left lung), but the sensitivity was limited (33.3% and 0%, respectively). This is likely due to the small number of patients with lung abscesses in our study and the challenges of detecting smaller or more complex lesions via ultrasound [25]. Similarly, for hydropneumothorax, LUS demonstrated high specificity (100%) but low sensitivity, consistent with the result of the study of Galetin et al. [26] who studied the accuracy of thoracic ultrasound for pneumothorax detection after lung resecting surgery and found that the sensitivity and specificity of ultrasound compared to chest radiographs were 32% and 85%, respectively. Our results may reflect a problem of insufficient statistical power rather than an absolute deficiency of the LUS technology itself.

Our findings suggest that pleural thickness, as measured by LUS, could predict the need for surgical intervention. A pleural thickness cut-off of >2.2 mm was associated with a higher likelihood of requiring continuous thoracic drainage and ultimately decortication. This is consistent with the study performed by Ehab F. Salim et al. [27] where the cutoff value at 3.95 mm was set for decortication via open thoracotomy with values less than this decortication was done also, but via thoracoscopic procedures. Although this cut-off differs from the 10 mm proposed in the study of Ionescu et al. [11]. Differences in cut-off values may be attributed to variations in patient population and methodologies; given the fact that in Ionescu et al. [11] the *pleural effusion thickness* was measured rather than the pleural thickness.

There is a statistically significant P value between the first and follow-up LUS (p value ≤ 0.05) regarding consolidation, collapse, presence of septations, pleural thickness, and the presence of effusion. Notably, follow-up LUS after one month showed significant improvement in pleural and parenchymal lung lesions. This is consistent with findings from other studies such as the study of Eid et al. [28] on patients who were clinically suspected of having pneumonia and serial follow up LUS showed gradual improvement in lung consolidation and effusion. Also Ionescu et al. [11] in their study performed follow up chest sonar after 5 days from the initiation of treatment and found improvement of consolidation in 46.7% and also improvement in pleural effusion which occurred in 55.6% of the cases. Our results support the use of LUS as a valuable tool for monitoring disease resolution and guiding management decisions.

In the current study, the P value is not significant between the first and follow-up LUS (P value is 1.000) regarding lung abscess and hydropneumothorax; this may be due to the small sample size or the fact that the lung sonar has low sensitivity in detection of both. To our knowledge, no available studies used LUS in the follow-up of both.

There remains a paucity of studies specifically examining the role of LUS in the follow-up of pediatric CAP and CCAP. Future research should focus on optimizing LUS protocols, training, and standardization to maximize its diagnostic and prognostic utility. Additionally, further studies are needed to explore the predictive role of LUS features, such as pleural thickness and aeration patterns, in guiding therapeutic interventions and predicting clinical outcomes.

The current study had some limitations including being a single-center study with limited number of participants. This has influenced the sensitivity of LUS for abscesses and hydropneumothorax which is insufficiently explored.

## Conclusion

Our study demonstrates that LUS is a highly sensitive and specific tool for detecting pleural effusion and lung consolidation in pediatric patients with CCAP. It offers a non-invasive, radiation-free alternative to CT, particularly suitable for resource-limited settings. However, its limitations in detecting certain pathologies, such as lung abscesses and pneumothorax, underscore the need for careful interpretation and, where necessary, complementary imaging. The utility of LUS in predicting the need for surgical intervention and monitoring disease progression further supports its role in managing pediatric CCAP. Future research should continue to refine LUS protocols and explore its potential in diverse clinical settings.

## Data Availability

All data used during the current study are available from corresponding author upon reasonable request.
